# A five-year model to assess the early cost-effectiveness of new diagnostic tests in the early diagnosis of rheumatoid arthritis

**DOI:** 10.1186/s13075-016-1020-3

**Published:** 2016-06-10

**Authors:** Leander R. Buisman, Jolanda J. Luime, Mark Oppe, Johanna M. W. Hazes, Maureen P. M. H. Rutten-van Mölken

**Affiliations:** Institute of Health Policy and Management, Erasmus University Rotterdam, PO Box 1738, 3000 DR Rotterdam, The Netherlands; Institute for Medical Technology Assessment, Erasmus University Rotterdam, PO Box 1738, 3000 DR Rotterdam, The Netherlands; Department of Rheumatology, Erasmus MC, University Medical Center Rotterdam, Rotterdam, The Netherlands; EuroQol Research Foundation, Rotterdam, The Netherlands

**Keywords:** Rheumatoid arthritis, Diagnosis, Treatment, Tests, Early cost-effectiveness analysis

## Abstract

**Background:**

There is a lack of information about the sensitivity, specificity and costs new diagnostic tests should have to improve early diagnosis of rheumatoid arthritis (RA). Our objective was to explore the early cost-effectiveness of various new diagnostic test strategies in the workup of patients with inflammatory arthritis (IA) at risk of having RA.

**Methods:**

A decision tree followed by a patient-level state transition model, using data from published literature, cohorts and trials, was used to evaluate diagnostic test strategies. Alternative tests were assessed as add-on to or replacement of the ACR/EULAR 2010 RA classification criteria for all patients and for intermediate-risk patients. Tests included B-cell gene expression (sensitivity 0.60, specificity 0.90, costs €150), MRI (sensitivity 0.90, specificity 0.60, costs €756), IL-6 serum level (sensitivity 0.70, specificity 0.53, costs €50) and genetic assay (sensitivity 0.40, specificity 0.85, costs €750). Patients with IA at risk of RA were followed for 5 years using a societal perspective. Guideline treatment was assumed using tight controlled treatment based on DAS28; if patients had a DAS28 >3.2 at 12 months or later patients could be eligible for starting biological drugs. The outcome was expressed in incremental cost-effectiveness ratios (€2014 per quality-adjusted life year (QALY) gained) and headroom.

**Results:**

The B-cell test was the least expensive strategy when used as an add-on and as replacement in intermediate-risk patients, making it the dominant strategy, as it has better health outcomes and lower costs. As add-on for all patients, the B-cell test was also the most cost-effective test strategy. When using a willingness-to-pay threshold of €20,000 per QALY gained, the IL-6 and MRI strategies were not cost-effective, except as replacement. A genetic assay was not cost-effective in any strategy. Probabilistic sensitivity analysis revealed that the B-cell test was consistently superior in all strategies. When performing univariate sensitivity analysis for intermediate-risk patients, specificity and DAS28 in the B-cell add-on strategy, and DAS28 and sensitivity in the MRI add-on strategy had the largest impact on the cost-effectiveness.

**Conclusions:**

This early cost-effectiveness analysis indicated that new tests to diagnose RA are most likely to be cost-effective when the tests are used as an add-on in intermediate-risk patients, and have high specificity, and the test costs should not be higher than €200–€300.

**Electronic supplementary material:**

The online version of this article (doi:10.1186/s13075-016-1020-3) contains supplementary material, which is available to authorized users.

## Background

Rheumatoid arthritis (RA) is a chronic inflammatory joint disease characterised by structural irreversible joint damage, leading to severe disability, serious loss of quality of life and premature death if left untreated [1–6]. Disease progression can be slowed down by synthetic and biologic disease-modifying antirheumatic drugs (DMARDs), especially if started early in the disease [7–11]. This requires early detection of RA. However, early diagnosis is complex, because RA-related symptoms early in the disease course resemble those of other musculoskeletal disorders [12]. Early detection would result in DMARDs being started early and improved prognosis. Therefore, several diagnostic tests are currently being developed to improve the early diagnosis of RA in patients with inflammatory arthritis (IA), e.g., B-cell related gene expression, IL-6 serum level test, magnetic resonance imaging (MRI) of hands and feet, and genetic assays with susceptibility single nucleotide polymorphisms (SNPs) for RA. However, little is known about their potential cost-effectiveness.

To guide implementation of new diagnostic tests in the workup of patients at risk of having RA, a decision model could be used to evaluate the test performance (i.e., sensitivity and specificity), test costs, and positioning of the test in terms of the clinical outcomes and societal costs. Evaluating the cost-effectiveness of new drugs before entering the market is well-implemented because reimbursement authorities request this. However, it is less common to evaluate the cost-effectiveness of new medical tests that enter the market while this also affects healthcare spending directly. Given the constraints on healthcare budgets, it is likely that clinicians evaluate the impact of new tests on their departmental budget and consider diagnostic uncertainty. To inform this clinical decision problem, we conducted an early cost-effectiveness analysis (early-CEA). In this analysis, the incremental costs of the new potential and current diagnostic test strategies are weighed against the gain in quality-adjusted life years (QALYs) and potential improved labour force participation [13]. The main assumption is that early diagnosis results in a timely start of effective treatment that reduces disease activity and consequently postpones or prevents treatment with a biologic DMARD.

The aim of this study was twofold. The first objective was to develop an early-CEA model to evaluate the costs and health effects (in terms of QALYs) of new and current diagnostic test strategies from a societal perspective in patients with IA who are suspected of having RA. The second objective was to analyse the costs and health effects of new test strategies compared to the American College of Rheumatology/European League Against Rheumatism (ACR/EULAR) 2010 RA classification criteria (referred to as RA-2010 criteria).

## Methods

We applied the framework following the general steps used in early-CEA of medical tests as developed by Buisman et al. [14]. This framework is a useful guidance for researchers performing early-CEA of medical tests. Early-CEA evaluates medical tests in development by assessing how much these tests could improve health outcomes and healthcare efficiency. Moreover, early-CEA helps test developers to decide about further development of medical tests, set realistic performance-price goals and design and manage reimbursement strategies [14, 15].

### Current diagnostic test strategy

IA patients at risk of having RA undergo a diagnostic workup to establish the presence of RA. This entails affected joint counts, blood testing, and radiographs ordered or established by the rheumatologist. If no other explanation for the symptoms is found (e.g., systemic lupus erythematosus (SLE), gout, or psoriatic arthritis) the patient is classified as having RA if at least 6 out of 10 points on the RA-2010 criteria are scored. Patients who score less than 6 but more than 2 points are regarded as patients at intermediate risk who do not fulfil the RA-2010 criteria. The RA-2010 criteria were the comparator in our early-CEA [6].

### New diagnostic test strategies

The cost-effectiveness was assessed of four diagnostic tests that are currently being developed as part of the TRACER project [16]: B-cell related gene expression [17], IL-6 serum level test [18], MRI of the hands and feet [19–24] and genetic assays with susceptibility SNPs for RA [25]. For each of the tests (described subsequently), three different test strategies were modelled: add-on to the RA-2010 criteria for all patients with IA, add-on for intermediate-risk patients only and replacement of all blood tests and radiographs used to classify patients according to the RA-2010 criteria. For the add-on test strategies, the performance of the new test strategy was estimated by combining the sensitivity and specificity of the RA-2010 criteria and the new tests.

#### B-cell related gene expression

During the development of arthritis, B-cell RNA expression decreases over time [16]. Although the exact mechanism is poorly understood, the marker is useful to predict early arthritis in patients with seropositive arthralgia [17]. Currently no data are available for patients with IA. Therefore, we used the data from the seropositive arthralgia cohort studied by Baarsen et al. [17]. After discussion with the developers of this test, sensitivity of 0.60 and specificity of 0.90 was used. The cost of the test was set at €150.

#### IL-6 serum level test

IL-6 is a cytokine that is present in inflammation. In a recent evaluation of IL-6 serum level test performance in detecting RA in patients with IA the sensitivity was 0.70 and specificity was 0.53 [18]. We used these sensitivity and specificity values and assumed a cost of €50 per test.

#### MRI of hands and feet

MRI may reclassify patients to different joint domains of the RA-2010 criteria if there are more swollen joints than are identified on physical examination. MRI also provides additional information on bone marrow edema [19] and tenosynovitis [20, 21]. Based on the literature [22–24] and discussions with test developers, we set the sensitivity of MRI at 0.90 and the specificity at 0.60. The costs of MRI were assumed to be equal to the unit costs currently used by the Dutch Healthcare Authority, of €189 per MRI examination (€756 for four MRI scans (both hands and both feet)) [26].

#### Genetic assay with susceptibility SNPs for RA

RA is a complex disease involving several genes. Heritability for RA is estimated to be around 50–60 % [25]. Expert review of the literature suggests that using genetic risk factors combined with current knowledge would result in sensitivity of 0.40 and specificity between 0.80 and 0.90 to identify patients with RA [25]. We used these estimations and applied sensitivity of 0.40 and specificity of 0.85 with an estimated cost of €750 per test based on expert opinion from test developers.

### Treatment

In the current and new diagnostic test strategies, test-positive patients received methotrexate (MTX) at 25 mg/week orally [27]. Due to the side effects of MTX, patients could switch to other synthetic DMARDs (e.g., Sulfasalazine, Leflunomide). After failure of two synthetic DMARDs, patients could switch to biologic DMARDs (i.e., TNF-inhibitors, IL6-inhibitors, B cell depletion, or T cell inhibition) [28].

RA patients who were additionally detected by the new diagnostic test strategies as compared to the RA-2010 criteria were assumed to be given early treatment. As a result, we modelled that they had an improvement of 0.2 in the disease activity score in 28 joints (DAS28) at 12 months as compared to patients in the current test strategy. The improvement of 0.2 in the DAS28 was based on sensitivity analysis in which we evaluated the effect of changing this value on the model results (see univariate sensitivity analysis below).

### Model structure

In RA, the diagnosis and subsequent prognosis are complex processes in which various tests and measures of disease activity influence treatment decisions and subsequently outcomes in terms of both costs and effects. As the diagnosis is often reconsidered in the first year of disease, especially in those initially not classified as having RA, we decided to model the first year as a decision tree with chance nodes at 6 and 12 months to classify patients as true positive (TP), false positive (FP), true negative (TN) and false negative (FN) during the first year. Patients were classified as TP if they had a positive test result (≥6 points on the RA-2010 criteria or positive on the new test) at baseline and at 12 months, used MTX or stopped MTX due to side effects. Moreover, the symptoms should not be explained by another classified diagnosis [6]. Patients were considered as TN if they had a negative test result (<6 points on the RA-2010 criteria or negative on the new test) at baseline, did not use MTX at 12 months, and had symptoms explained by another classified diagnosis. Patients were considered FP if they scored ≥6 points on the RA-2010 criteria or were positive on the new test at baseline but did not use MTX at 12 months, and had symptoms explained by another classified diagnosis. Patients were classified as FN if they scored <6 points on the RA-2010 criteria or were negative on the new test at baseline but used MTX or stopped MTX due to side effects at 12 months, and had no symptoms explained by another classified diagnosis. Using a combination of initial RA-2010 criteria scores and the use of DMARDs not explained by any other disease is a common way of dealing with a disease for which no hallmark sign is available [6, 29].

**Fig. 1:**
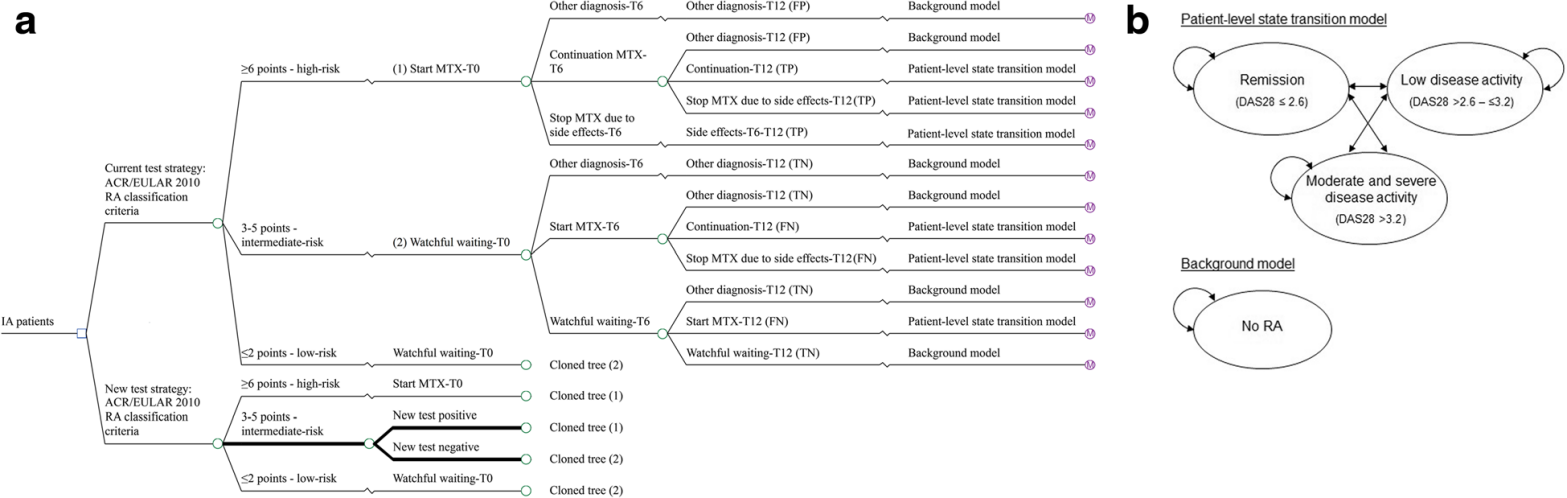
**a** Decision tree for the first year comparing the current diagnostic test strategy with the new diagnostic add-on test strategy for intermediate-risk patients. *Current test strategy* American College of Rheumatology/European League Against Rheumatism (*ACR/EULAR*) 2010 rheumatoid arthritis (RA) classification criteria. *New test strategy* add-on test for intermediate-risk patients (3–5 points) according to the ACR/EULAR 2010 RA classification criteria. *IA* inflammatory arthritis, *MTX* methotrexate, *TP* true positive, *FP* false positive, *TN* true negative, *FN* false negative, *T0* baseline, *T6* 6 months, *T12* 12 months. **b** Patient-level transition state model and background model of second to fifth year for all test strategies. *DAS28* disease activity score in 28 joints

The first year is followed by a four-year individual-level Markov model (i.e., patient-level state transition model) that simulates the change in disease activity (DAS28) over time in 3-month cycles. The cycle time is 3 months because patients are commonly seen by the rheumatologists every 3 months. This 5-year model was used to simulate what would happen if a proportion of FN patients in the current strategy were diagnosed earlier with lower levels of disease activity in the new test strategy. The time horizon was 5 years because the long-term effects of biological drug use are unknown. Patients were categorised into three disease states: remission (DAS28 ≤ 2.6), low disease activity (DAS28 > 2.6 to ≤3.2) and moderate and severe disease activity (DAS28 > 3.2).^1^ This categorization by DAS28 score is common in the field of RA [30–32]. Resource use, costs and utilities were linked to these three categories. The patients who were classified as TP or FN at 12 months entered the patient-level state transition model. A proportion of patients with DAS28 > 3.2 were modelled to start a biologic DMARD in addition to MTX. They were assumed to stay on a biologic DMARD and could switch to another biologic DMARD for the remainder of the 4 years. The patients who were classified as TN or FP at 12 months entered a background model in which they stayed for the remaining 4 years, assuming no change in utilities, biologic DMARD costs for 10 % of FP patients in the first year after diagnosis and otherwise, no RA-related costs. Figure 1 shows the decision model comparing the current diagnostic test strategy with the new add-on diagnostic test strategy for intermediate-risk patients (described subsequently). This 5-year cost-effectiveness model is an extension of our 1-year model published elsewhere [33].

### Data sources

To populate the model, we mainly used data from three different sources. Additional file 1: Table S1 shows the characteristics of the three sources. First, data from the REACH cohort (usual care) were used to populate the 1-year decision tree with 552 patients with IA who were suspected of having RA (details about the cohort can be found in Additional file 2: Table S2). Patients had to have at least one joint clinically diagnosed as affected by synovitis that could not be classified as another inflammatory joint disease. The prevalence of RA was 54 % at 12 months based on the RA-2010 criteria and MTX use.

Second, data from the tREACH trial were used in our model for RA patients after 1-year follow-up [34]. The tREACH trial includes patients aged 18 year-old or older with arthritis in at least one joint, and symptom duration less than 1 year. Patients were randomized into three initial treatment strategies: triple DMARD therapy (MTX, sulfasalazine and hydroxychloroquine) with intramuscular glucocorticoids, triple DMARD therapy with an oral glucocorticoid tapering scheme and MTX monotherapy with an oral glucocorticoid tapering scheme. See Claessen et al. [35] for a detailed description of the tREACH trial.

Third, summary data from the DREAM registry as published by Vermeer et al. [36] were used to inform on the start of biological drugs. This publication describes data from two cohorts, a treat-to-target cohort and a usual-care cohort of patients with clinical RA. The treat-to-target strategy used a standardized treatment step-up protocol [36]. In contrast, the treatment switches were not performed by protocols in the usual care cohort.

### Model inputs

Additional file 3: Table S3 gives an overview of all model input parameters, their estimates and distributions for probabilistic sensitivity analysis, and data sources.

### Estimation of transition probabilities

During the first 12 months of our model, the probabilities of patients being TP, FN, TN and FP were elicited from the REACH cohort in which patients were classified according to the RA-2010 criteria, use of MTX and use of other synthetic DMARDs at baseline, 6 and 12 months.

At the start of the patient-level state transition model (i.e., at 12 months), the DAS28 of TP and FN patients at 12 months in the REACH cohort resulted in patients entering one of the three disease states. Patients who entered the DAS28 >3.2 state at the start or later in time could be eligible for starting biological drugs. To model this, summary data from the DREAM cohort on the start of biological drugs were used, in which the observed use of biologic DMARDs in clinical practice was 15 % at 24 months. We transformed this 15 % rate into a 3-month transition probability of 2 % to start biologic DMARDs in those patients with a DAS28 >3.2. This 2 % was distributed over the three disease states in a 1–3–6 distribution (state 1– state 2– state3) based on flare rates (DAS28 > 3.2) in the tREACH cohort.

### Estimates of resource use and costs

We distinguished two cost categories: direct medical and productivity costs. Direct medical costs include costs of visits to rheumatologists and other health professionals (e.g., physical therapist), laboratory tests including diagnostic tests and those to monitor side effects, and medication use. Productivity costs represent the number of days that a patient with a paid job was absent from work in the past 3 months. Resource use and productivity losses per disease state were obtained from the REACH study in the first year [29] and from the tREACH study [34, 35] in the second and third year. The latter was extrapolated to 5 years. In the background model TN patients were assumed to incur no RA-related costs, while 10 % of FP patients incurred biologic DMARD costs in the first year after diagnosis due to misdiagnosis, for which the frequency was based on the REACH study.

The unit costs of visits and productivity losses were based on reference prices published in the Dutch Manual of Costing in economic evaluations [37]. Diagnostic test costs were based on tariffs published by the Dutch Healthcare Authority [26], and medication costs were obtained from the National Health Care Institute [38]. All costs were adjusted to €2014 using the general price index from the Dutch Central Bureau of Statistics [39]. All cost parameters can be found in Additional file 3: Table S3.

### Estimation of QALYs

When assessing the impact of a new test or treatment on quality of life over time, the health outcomes are usually measured in terms of quality-adjusted life years (QALYs). The QALY combines the number of life years with the level of health-related quality of life (i.e., utilities) in those years [40]. The EuroQol 5-dimension 3-level questionnaire (EQ-5D-3L) was used to estimate utilities. The baseline utilities of TP, FP, TN, and FN were obtained from the REACH study and were 0.60, 0.65, 0.65 and 0.60, respectively. Based on the literature we assigned an improvement of 0.10 over the first year to the TPs [41–45]. Based on the REACH study we assigned an improvement of 0.05 and 0.10 over the first year to the FPs and TNs, respectively. Based on the placebo group in the STIVEA trial, we assigned a 0.05 reduction over the first year for FNs, assuming that FN patients would receive little therapy [45].

In the patient-level state transition model, patients were assigned EQ-5D values based on their DAS28 every 3 months, stratified for the start of biologic DMARDs. As observed in the tREACH study, the EQ-5D values for patients not using biologic DMARDs were higher. Furthermore, the EQ-5D values were not normally distributed. About 25 % of patients in the tREACH study had a decrease in EQ-5D at least once in 3 years, which led to a utility score lower than 0.50. Therefore, different distributions of utility values were estimated. One distribution was estimated for patients with at least one EQ-5D decrease below 0.50 over time and another distribution was estimated for patients who always had an EQ-5D higher than 0.50 over time. In the background model, patients were assumed to have an EQ-5D value of 0.75 that remained constant over time.

### Analyses/modelling

We performed a base-case analysis with four diagnostic tests that were used in three diagnostic test strategies as described above. We calculated the incremental costs per QALY gained in each new test strategy compared with the current test strategy (i.e., incremental cost-effectiveness ratio (ICER)). Probabilistic sensitivity analyses were performed in which incremental costs and QALYs were calculated as the mean of 1,000 Monte Carlo simulations, where each simulation samples simultaneously from the appropriate distributions of the input parameters (see Additional file 3: Table S3 for the distributions). Cost-effectiveness planes and acceptability curves were constructed from the Monte Carlo simulation. In addition, we used the headroom (i.e., potential profit) method to assess the maximum additional cost for which each new diagnostic test was still likely to be cost-effective at a willingness-to-pay threshold of €20,000 per QALY gained [46, 47].

Furthermore, we explored the impact of our model parameters in univariate sensitivity analyses, varying the sensitivity, specificity, new test costs, improvement in the DAS28 in TP patients in the new test strategy, who were FN in the current test strategy, and costs of biologic DMARDs for FP patients in the first year after diagnosis. The range over which the model parameters were varied are shown between brackets in Fig. 4. We report these analyses for an add-on test for intermediate-risk patients. For each analysis, one model parameter was altered while the other parameters were held constant at the baseline value. In our analyses, differential discounting was applied in accordance with the Dutch guidelines for economic evaluation research, with an annual discount rate of 4.0 % for all costs and 1.5 % for health effects [48].

### Model validation

The model structure and input parameters were checked for clinical correctness by rheumatologists. We also verified the model for coding and logical correctness by running extreme value scenarios. Furthermore, an independent modeller internally validated our model to check the model structure, all input parameters with distributions and the visual basic code used to programme the model in Excel.

### Ethical approval and patient consent

No ethical approval and consent from patients was needed for this study.

## Results

### Reclassification

In the add-on test strategy, only intermediate-risk patients could be reclassified as high risk or low risk of having RA, and low-risk patients could be reclassified as high-risk patients. Table 1 shows the reclassification for both add-on strategies. Due to the high specificity of the B-cell test and genetic assay, more patients were reclassified as low risk, while due to the high sensitivity of IL-6 and MRI, more patients were reclassified as high risk.

**Table 1:** Reclassification table with the results for intermediate-risk patients according to the ACR/EULAR 2010 RA classification criteria and for all patients

	Number of patients according to ACR/EULAR 2010 RA classification criteria	Number of patients reclassified as high risk	Number of patients reclassified as low risk	Combined sensitivity: RA-2010 criteria + new test	Combined specificity: RA-2010 criteria + new test
(A) Add-on test for all patients					
B cell test				0.85	0.69
High risk	243	243	0		
Intermediate risk	263	75 (29 %)	188 (71 %)		
Low risk	46	13 (28 %)	33 (72 %)		
IL-6 test				0.89	0.41
High risk	243	243	0		
Intermediate risk	263	146 (56 %)	117 (44 %)		
Low risk	46	26 (57 %)	20 (43 %)		
Magnetic resonance imaging				0.96	0.46
High risk	243	243	0		
Intermediate risk	263	154 (59 %)	109 (41 %)		
Low risk	46	26 (59 %)	20 (41 %)		
Genetic assay test				0.77	0.66
High risk	243	243	0		
Intermediate risk	263	64 (24 %)	199 (76 %)		
Low risk	46	11 (24 %)	35 (76 %)		
(B) Add-on test for intermediate-risk patients					
B cell test				0.81	0.71
High-risk	243	243	0		
Intermediate risk	263	75 (29 %)	188 (71 %)		
Low risk	46	0	46		
IL-6 test				0.85	0.46
High risk	243	243	0		
Intermediate risk	263	146 (56 %)	117 (44 %)		
Low risk	46	0	46		
MRI				0.91	0.51
High risk	243	243	0		
Intermediate risk	263	154 (59 %)	109 (41 %)		
Low risk	46	0	46		
Genetic assay test				0.75	0.67
High risk	243	243	0		
Intermediate risk	263	64 (24 %)	199 (76 %)		
Low risk	46	0	46		

Combined sensitivity = sensitivity of American College of Rheumatology/European League Against Rheumatism (ACR/EULAR) 2010 rheumatoid arthritis (RA) classification criteria + sensitivity of the new test. Combined specificity = specificity of ACR/EULAR 2010 RA classification criteria + specificity of the new test.

### Cost-effectiveness

Table 2 shows the results of the RA-2010 criteria and the four new tests used as add-on for all patients, add-on for intermediate-risk patients, and replacement of the RA-2010 criteria.

**Table 2:** Five-year cost-effectiveness of new test strategies versus current test strategy

Test strategy	New test	Se^a^	Sp^a^	Test costs^a^	TP *n* (%)	FP *n* (%)	TN *n* (%)	FN *n* (%)	Costs	QALYs	∆Costs	∆QALYs	ICER	Headroom^b^
ACR/EULAR 2010 RA		0.62	0.77	€1,593^d^	185 (34)	58 (11)	195 (35)	114 (21)	€16,784	3.430				
Add-on all patients	B-cell	0.60	0.90	€150	254 (46)	77 (14)	175 (32)	46 (8)	€16,807	3.454	€23	0.024	€969	€602
	IL-6	0.70	0.53	€50	265 (48)	149 (27)	103 (19)	34 (6)	€17,387	3.451	€603	0.021	€28,171	-€125^e^
	MRI	0.90	0.60	€756	288 (52)	135 (24)	117 (21)	11 (2)	€17,848	3.461	€1,063	0.031	€34,318	€312
	Genetic	0.40	0.85	€750	231 (42)	87 (16)	166 (30)	69 (13)	€17,611	3.444	€827	0.014	€57,606	€210
Add-on intermediate-risk patients	B-cell	0.60	0.90	€150	244 (44)	74 (13)	178 (32)	56 (10)	€16,748	3.450	-€37	0.020	Dominant^c^	€511
	IL-6	0.70	0.53	€50	254 (46)	135 (24)	117 (21)	46 (8)	€17,271	3.448	€487	0.018	€26,696	-€72^e^
	MRI	0.90	0.60	€756	273 (49)	124 (22)	128 (23)	27 (5)	€17,404	3.456	€620	0.026	€23,457	€269
	Genetic	0.40	0.85	€750	224 (41)	82 (15)	170 (31)	75 (14)	€17,211	3.442	€427	0.012	€35,233	€173
Example of what would happen if one would replace the RA classification criteria														
Replacement all	B-cell	0.60	0.90	€150	180 (33)	25 (5)	227 (41)	120 (22)	€15,983	3.432	-€801	0.002	Dominant^c^	€984
	IL-6	0.70	0.53	€50	210 (38)	119 (22)	134 (24)	90 (16)	€16,849	3.434	€64	0.004	€17,526	€59
	MRI	0.90	0.60	€756	270 (49)	101 (18)	151 (27)	30 (5)	€17,139	3.458	€355	0.027	€12,906	€951
	Genetic	0.40	0.85	€750	120 (22)	38 (7)	214 (39)	180 (33)	€16,675	3.414	€ -110	-0.016	€6,914	€543

*Se* sensitivity, *Sp* specificity, *TP* true positive, *FP* false positive, *TN* true negative, *FN* false negative, *QALY* quality-adjusted life year, *ICER* incremental cost-effectiveness ratio (€ per QALY gained), *ACR/EULAR* American College of Rheumatology/European League Against Rheumatism, *RA* rheumatoid arthritis, *MRI* magnetic resonance imaging. ^a^These are the Se, Sp and costs of the new test; the Se and Sp of the combination of the new test plus the ACR/EULAR criteria are reported in the text. ^b^Willingness to pay threshold is €20,000 per QALY gained. ^c^Dominant = better health outcomes and lower costs. ^d^Costs of visits and diagnostic tests during first year. ^e^Mainly due to the low specificity, an IL-6 test as an add-on can never be cost-effective compared to the current test strategy

The B-cell test was the least expensive strategy when used as an add-on in intermediate-risk patients and as a replacement test, making it the dominant strategy, as it has better health outcomes and lower costs. The B-cell test was also the most cost-effective test strategy as an add-on in all patients. When using a willingness-to-pay threshold of €20,000 per QALY gained, the IL-6 and MRI test strategies were not cost-effective, except in the replacement strategy. A genetic assay was not cost-effective in any strategy. When comparing the test strategies, replacement of the RA-2010 criteria was the most cost-effective test strategy, followed by the add-on test in intermediate-risk patients, and the least favourable was the add-on test in all patients.

### Headroom

Table 2 shows the maximum additional cost for which each new test was likely to be cost-effective at a willingness-to-pay threshold of €20,000 per QALY gained (i.e., the headroom). Given the sensitivity and specificity of the different tests, an IL-6 test will only be cost-effective with a unit cost below €59 in the replacement test strategy. The headroom of a genetic assay test (€210 as the add-on in all patients, €173 as the add-on in intermediate-risk patients, and €543 as the replacement) also shows that the current unit costs of this test were too high, because the headroom was lower than the current unit costs of this test (€750).

### Probabilistic sensitivity analysis

**Fig. 2:**
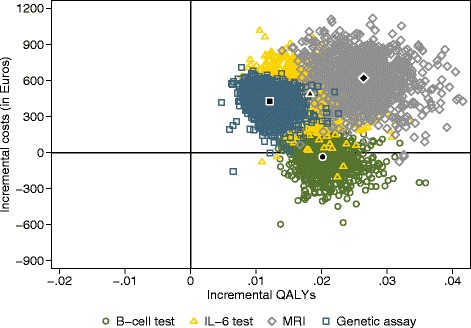
Cost-effectiveness planes of add-on for the intermediate-risk test strategy versus the current diagnostic test strategy. Current test strategy = American College of Rheumatology/European League Against Rheumatism (ACR/EULAR) 2010 rheumatoid arthritis (RA) classification criteria. *QALY* quality-adjusted life year, *MRI* magnetic resonance imaging

Figure 2 shows the cost-effectiveness planes with the average costs and effects and the uncertainty around this average for the add-on test strategy in intermediate-risk patients. All estimates lie within the northeast or southeast quadrants, meaning improved health outcomes. In the northeast quadrant, this is accompanied by higher costs as seen for IL-6, MRI, and gene assay. Nearly 100 % of these estimates (99.6 %, 99.7 %, and 99.8 %, respectively) lie within this quadrant. For the B cell test, part of the estimate (57.2 %) lies within the southeast quadrant depicting lower costs and improved health outcomes. Uncertainty for the MRI and IL-6 test were greater than for the B cell test and gene assay as shown by the width and height of the cloud, which is a consequence of the low specificity of 0.60 (MRI) and 0.53 (IL-6).

**Fig. 3:**
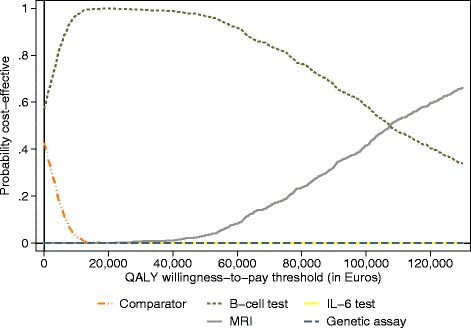
Cost-effectiveness acceptability curves for add-on for the intermediate-risk test strategy and the current diagnostic test strategy. Current test strategy = American College of Rheumatology/European League Against Rheumatism (ACR/EULAR) 2010 rheumatoid arthritis (RA) classification criteria. *QALY* quality-adjusted life year, *MRI* magnetic resonance imaging

Figure 3 shows the cost-effectiveness acceptability curves for the add-on test strategy for intermediate-risk patients. If a willingness-to-pay threshold of €20,000 per QALY gained is used, there is a probability of cost-effectiveness for the B-cell test of 100 %.

### Univariate sensitivity analysis

The sensitivity of our results was evaluated by varying single model parameters and comparing the ICERs to the base-case ICERs of the most cost-effective test (B cell) and the most effective test (MRI) when used as the add-on in intermediate-risk patients. The base-case ICERs were estimated using an improvement of 0.2 in the DAS28 at 12 months in the FN patients, 10 % of FP patients with biologic DMARD costs in the first year after diagnosis and the test-specific sensitivity, specificity and costs.

**Fig. 4:**
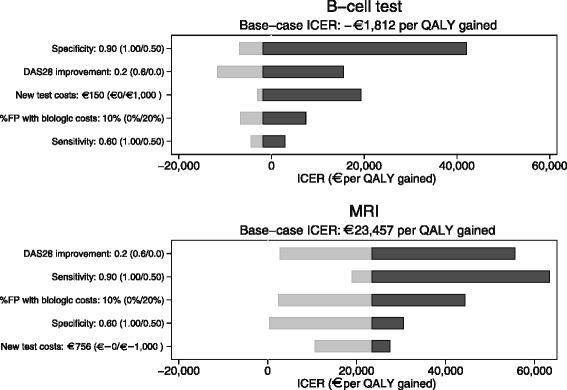
Impact of varying model inputs on incremental cost-effectiveness ratio for an add-on B-cell test or magnetic resonance imaging (*MRI*) in intermediate-risk patients. *QALY* quality-adjusted life year, *ICER* incremental cost-effectiveness ratio, *FP* false positive

For a B-cell test, the change in specificity had the largest impact (see Fig. 4), followed by improvement in the DAS28 in TP patients in the new test strategy, who were FN in the current test strategy. No improvement in the DAS28 resulted in an ICER of about €16,000, while an improvement of 0.6 in the DAS28 resulted in a dominant new test strategy (i.e., better health outcomes and lower costs). Varying the new test costs, the number of FP patients having biological DMARD costs between 0 % and 20 %, and sensitivity had less impact on the ICER, but still caused variation.

For MRI, the change in improvement in the DAS28 in TP patients in the new test strategy, who were FN in the current test strategy, had the largest impact (see Fig. 4). No improvement in DAS28 resulted in an ICER of about €56,000, while an improvement of 0.6 in the DAS28 resulted in an ICER of about €3000. Changing sensitivity to 0.50 or 1.00 had about the same impact as varying the number between 0 % and 20 % of FP patients having biological DMARD costs. Adjustments in specificity and costs had the least impact on the ICER.

## Discussion

Various new medical technologies enable the detection of RA at an increasingly earlier stage. RA is a disease in which early detection has high potential because there are treatments available that effectively reduce disease progression, especially if introduced early in the disease. In our study, we compared four different tests and we found that a B-cell test was the most cost-effective test in all test strategies. This is mainly due to the high specificity (0.90) in combination with moderate sensitivity (0.60), and relatively low test costs (€150). When the specificity of the test increases, the number of FP patients who may receive unnecessary expensive treatment is reduced, which largely increases the likelihood that the test becomes cost-effective. MRI was the second most cost-effective test as an add-on in intermediate-risk patients. Mainly as a result of the higher costs of MRI (€756) compared to a B-cell test (€150), MRI was less cost-effective, even though it had much higher sensitivity than the B-cell test (0.90 versus 0.60). However, the MRI had lower specificity, which had more impact on the ICER than lower sensitivity. The specificity is more important because an add-on test with a specificity below 100 % results in more patients classified as FP, while these patients were TN according to the current test strategy.

This study indicated where it is most likely to position a new diagnostic test given the sensitivity, specificity and costs in relation to the current RA-2010 criteria test strategy. B-cell gene expression as an add-on test in intermediate-risk patients was the most likely cost-effective add-on strategy and could even replace the RA-2010 criteria, given its moderate sensitivity (0.60), high specificity (0.90) and low costs (€150). However, it is unlikely that a replacement test would be discovered that provides the rheumatologists with the same richness of information as the current RA-2010 criteria. To use a new test in addition to the RA-2010 criteria in all patients is the least cost-effective, because it would not alter treatment for the high-risk patients (243 of 552 patients). These patients will have higher test costs without additional health gain.

The discussion about the potential benefits of early detection and treatment of RA is also relevant in the light of the current discussion about biosimilar drugs. Currently, the price of biologic DMARDs is set at €14,000 per patient per year. Due to this high price (which is about the same for all biologic DMARDs), it is an important driver of the cost-effectiveness results. If the price of a biosimilar will be significantly lower, it will have a smaller impact on the results. On the one hand, this could result in starting biological drugs earlier, resulting in more TP patients using biologic DMARDs. On the other hand, a cheaper biosimilar may worsen the cost-effectiveness of new tests because the savings from postponing or preventing treatment with a biologic drug are smaller. The net result is hard to predict, as prescription behaviour may change with lower costs of biological drugs.

The results of our study are likely to be generalizable to other countries and different healthcare systems because the RA-2010 criteria are widely used internationally for classifying RA. The costs of diagnostic tests and treatment patterns obviously differ between countries and healthcare systems (e.g., higher costs in the USA). However, this would not result in differences in the most cost-effective test strategy.

The results of our simulation should be interpreted taking into account that we used fixed values for the sensitivity, specificity and costs of the alternative tests. This was done to show the differences in early cost-effectiveness between tests with different test characteristics. If we had added distributions around these parameters, the differences in cost-effectiveness between the tests would likely decrease due to large uncertainties of these parameters in early-CEA. To overcome this, we performed univariate sensitivity analysis to explore the impact of changing these parameters. We found that multiple factors had an impact on the cost-effectiveness of a new test strategy. The main drivers of the ICER include the sensitivity, specificity and costs of the new test, but also the improvement in the DAS28 for TP patients in the new test strategy, who were FN according to the RA-2010 criteria, and the percentage of FP patients with biological DMARD costs in the first year after diagnosis.

In addition, as RA is a heterogeneous disease, a new test might provide additional diagnostic information in particular subgroups, such as obese patients or those with coexistent osteoarthritis. Furthermore, the choice of a new test might also be guided by additional diagnostic information that is expected in particular subgroups. Further research should investigate the impact of this additional diagnostic information on the cost-effectiveness of these new tests.

Like any early-CEA study, our study had limitations. One was that limited data were available on long-term disease progression in a cohort receiving usual care. Therefore, we had to synthesize data from different data sources and to make assumptions about the new test strategies based on expert opinion. Typically, the improvement in health outcomes of adding a new test without observed data is difficult to estimate. In the first year after diagnosis, we assigned equal improvements in utilities for TP and TN patients, less gain for FP patients, and reduced utilities for FP patients. Whether this reflects clinical practice needs to be proven. Another limitation could be that a death state was not included in our model. However, because of the low (1–2 %) mortality risk observed in patients with RA during 5 years of follow up [49], and the equal mortality risk with the current and new diagnostic test strategy, the mortality risk would not influence the model results.

## Conclusions

We have shown that the B-cell gene expression as an add-on test for intermediate-risk patients was the most likely cost-effective add-on strategy. A new add-on test for intermediate-risk patients should have high specificity and the costs should not be higher than €200–€300.

## Additional files

Additional file 1: Table S1. Cohort characteristics. (PDF 181 kb) Additional file 2: Table S2. Characteristics of the unclassified patients with inflammatory arthritis in the REACH study. (PDF 164 kb) Additional file 3: Table S3. Model input parameters. (PDF 886 kb)

## Abbreviations

DAS: disease activity score DAS28, disease activity score in 28 joints
DMARD: disease-modifying antirheumatic drug
Early-CEA: early cost-effectiveness analysis
ACR: American College of Rheumatology
EQ-5D-3L: EuroQol five dimension 3-level questionnaire
EULAR: European League Against Rheumatism
FN: false negative
FP: false positive
IA: inflammatory arthritis
ICER: incremental cost-effectiveness ratio
IL: interleukin
MRI: magnetic resonance imaging
MTX: methotrexate
QALY: quality-adjusted life year
RA: rheumatoid arthritis
RA-2010 criteria: American College of Rheumatology/European League Against Rheumatism (ACR/EULAR) 2010 rheumatoid arthritis classification criteria
Se: sensitivity
SNP: susceptibility single nucleotide polymorphism
Sp: specificity
TN: true negative
TNF: tumour necrosis factor
TP: true positive

## Footnotes

1 Moderate and severe DAS28 were categorised in one disease state. As treatment choice is based on DAS28 >3.2, our categorisation does not affect the model results.
