# Supplementary material for: A five-year model to assess the early cost-effectiveness of new diagnostic tests in the early diagnosis of rheumatoid arthritis
Source: Arthritis Res Ther. 2016 Jun 10;18:135. doi: 10.1186/s13075-016-1020-3 (PMC4902954; doi:10.1186/s13075-016-1020-3)
Supplement: Additional file 1: Table S1. — Cohort characteristics. (PDF 181 kb) [file 13075_2016_1020_MOESM1_ESM.pdf]

Additional file 1: Table S1. Cohort characteristics

Source	Patient population	Age (SD)	Sex (% female)	ACCP/RF positive	≥6 points on RA-2010 criteria	Intervention	Control	QALY data available	Work outcomes data available
**REACH [1]**	Newly detected early inflammatory arthritis patients	53 (15)	68%	43%	42%	Usual care	NA	Yes	Yes
**tREACH [2]**	Newly diagnosed RA patients	I: 53 (15)<br>I: 54 (14)<br>C: 54 (14)	60%<br>72%<br>70%	81%/76%<br>72%/70%<br>77%/67%	97%	Methotrexate, Plaquenil, Sulphazalazine Prednisone as bridging therapy	Methotrexate, Prednisone as bridging therapy	Yes	Yes
**DREAM registry [3]**	Newly diagnosed RA-patients (clinical diagnosis)	I: 58 (14)<br>C: 57 (13)	61.7%	68%*	NG	Treat- to-target step-up therapy	Usual care	No	No

I: Intervention; C: Comparator; SD: standard deviation; QALY: quality-adjusted life year; *RF only; NG: Not Given; NA: Not Applicable.

References

[1] REACH data on file.

[2] De Jong PH, Hazes JM, Han HK, et al. Randomised comparison of initial triple DMARD therapy with methotrexate monotherapy in combination with low-dose glucocorticoid bridging therapy; 1-year data of the tREACH trial. Ann Rheum Dis 2014;73(7):1331–9.

[3] Vermeer M, Kievit W, Kuper HH, et al. Treating to the target of remission in early rheumatoid arthritis is cost-effective: results of the DREAM registry. BMC Musculoskelet Disord 2013;14:350.
