# Supplementary material for: A five-year model to assess the early cost-effectiveness of new diagnostic tests in the early diagnosis of rheumatoid arthritis
Source: Arthritis Res Ther. 2016 Jun 10;18:135. doi: 10.1186/s13075-016-1020-3 (PMC4902954; doi:10.1186/s13075-016-1020-3)
Supplement: Additional file 2: Table S2. — Characteristics of the unclassified patients with inflammatory arthritis in the REACH study. (PDF 164 kb) [file 13075_2016_1020_MOESM2_ESM.pdf]

Additional file 2: Table S2. Characteristics of the unclassified inflammatory arthritis patients in REACH

		All patients (n=552)
Age (in years; SD)		53 (15)
Sex (% female)		68%
**Domains of ACR/EULAR 2010 RA classification**		
Swollen Joints	> 10	19%
	4-10 small	34%
	1-3 small	33%
	2-10 large	3%
	1 large	6%
Serology	high	34%
	low	9%
Disease duration: >6 weeks		85%
Elevated acute phase reactants		53%
**Score on ACR/EULAR 2010 RA classification**		
6 or more points		42%
3-5 points		48%
0-2 points		11%

SD: standard deviation.

Design of the Rotterdam Early Arthritis CoHort

The Rotterdam Early Arthritis CoHort (REACH) is an inception cohort study with 2 years of follow up. Assessments took place at baseline, 6 months, 12 months and 24 months. REACH aims to study the etiopathogenesis, diagnostic strategies, and outcome of patients with inflammatory joint conditions for <12 months. Both general practitioners and rheumatologists invited patients to participate in REACH from July 2004 to April 2011. For general practitioners, short educational courses on the importance of early treatment of RA and early referral were organized. Physicians that agreed to participate in REACH received written information and verbal instructions on the general aims of the study and on how to

send patients for inclusion in the study. Data collection includes a large array of detailed medical examinations and questionnaires.

General practitioners selected patients with arthritis in ≥1 joint or patients experiencing conditions in ≥2 joints without synovitis. The general practitioners determined that conditions existed for <12 months and were not due to trauma/mechanical problems. In addition, patients had to be age >16 years. During an interview by telephone and a subsequent medical examination by a rheumatologist, the inclusion criteria were verified. Patients were included if 1) joint conditions existed for <12 months with no requirement of a minimum duration; 2) they had arthritis in ≥1 joint or conditions in ≥2 joints in combination with at least 2 of the following criteria ascertained during medical examination by a rheumatologist: morning stiffness for >1 hour, bilateral compression pain in the metacarpophalangeal or metatarsophalangeal joints, symmetric presentation, positive family history, non-fitting shoes, non-fitting rings, pins and needles in fingers, or unexplained fatigue for <1 year; and 3) conditions were predominantly present in the morning and at night, and improved with movement. Patients were excluded if 1) conditions were due to trauma/mechanical problems, 2) they were age <16 years, 3) no written communication was possible in Dutch, or 4) a prior diagnosis of RA, ankylosing spondylitis, Sjögren's syndrome, systemic lupus erythematosus, or juvenile arthritis had been made by a rheumatologist before inclusion in this study.

For patients directly visiting rheumatologists, a similar verification procedure was applied. The final diagnosis was made by a rheumatologist in one of the 5 participating out-patient rheumatology clinics.

The study was approved by the Erasmus MC Medical Ethical Committee. All patients gave written informed consent.

In the current study we use 552 consecutive patients with unclassified inflammatory arthritis.
