# Supplementary material for: A five-year model to assess the early cost-effectiveness of new diagnostic tests in the early diagnosis of rheumatoid arthritis
Source: Arthritis Res Ther. 2016 Jun 10;18:135. doi: 10.1186/s13075-016-1020-3 (PMC4902954; doi:10.1186/s13075-016-1020-3)
Supplement: Additional file 3: Table S3. — Model input parameters. (PDF 886 kb) [file 13075_2016_1020_MOESM3_ESM.pdf]

Additional file 3: Table S3. Model input parameters

Parameter	Value	SE	Distribution (alpha;beta)	Source
**Prevalence of RA based on ACR/EULAR 2010 RA classification criteria**				
All patients	0.540	0.021	Beta (300;252)	REACH+
Intermediate risk patients	0.370	0.030	Beta (97;166)	REACH+
**Probabilities**				
**Methotrexate start and continuation**				
Patients with ≥6 points at baseline				
Start at baseline	1.000			[1]
Continuation at 6 months	0.840	0.024	Dirichlet (1,282;0.0007)	REACH+
Continuation at 12 months†	0.880	0.026	Dirichlet (1,125;0.0008)	REACH+
<6 points at baseline				
Start at baseline	0.000			[1]
Start at 6 months	0.350	0.027	Dirichlet (167;0.0021)	REACH+
Continuation at 12 months†	0.840	0.035	Dirichlet (592;0.0014)	REACH+
Start at 12 months	0.140	0.053	Dirichlet (7;0.0199)	REACH+
**Other diagnosis**				
≥6 points at baseline				
6 months	0.140	0.022	Dirichlet (35;0.0038)	REACH+
12 months	0.120	0.021	Dirichlet (201;0.0045)	REACH+
<6 points at baseline				
6 months	0.510	0.028	Dirichlet (322;0.0016)	REACH+
12 months	0.000	0.000	Dirichlet (0.000; 0.000)	REACH+
Biological use at 12 months	0.100	0.021	Beta (0.027;21)	REACH+, DREAM [2],
Biological start – per 3 months				

Parameter	Value	SE	Distribution (alpha;beta)	Source
Total	0.020	0.010	Beta (4;48)	DREAM [2]
DAS28≤2.6	0.002			
DAS28>2.6–≤3.2	0.006			
DAS28>3.2	0.012			
EQ-5D always >0.5 over time				
Patients not using a biological	0.752	0.024	Beta (245;81)	tREACH++
Biological patients	0.736	0.030	Beta (155;56)	tREACH++
**DAS28 increase at time of biological start**				
Delta DAS28≤2.6	2.064	0.120	Normal	tREACH++
Delta DAS28>2.6–≤3.2	0.860	0.058	Normal	tREACH++
**OLS functions of DAS28 over time since start biological**				
0–3 months from biological start				
Time	-0.290	0.038	Normal	tREACH++
Constant	4.019	0.109	Normal	tREACH++
4–15 months from biological start				
Time	-0.044	0.014	Normal	tREACH++
Constant	3.564	0.172	Normal	tREACH++
16–48 months from biological start				
Time	-0.015	0.011	Normal	tREACH++
Constant	3.426	0.289	Normal	tREACH++
**Costs**				
**Costs during 1st year – 0-12 months**				
Baseline visit and diagnostic tests	€917	€138	Gamma (€44;€21)	Estimate***

Parameter	Value	SE	Distribution (alpha;beta)	Source
Follow-up visits and diagnostic tests during first year	€676	€101	Gamma (€44;€15)	Estimate***
MTX costs plus monitoring during first year	€102	€15	Gamma (€44;€2)	Estimate***
Other sDMARD costs				
True positive patients	€165	€10	Gamma (€253;€0.65)	REACH+
False positive patients	€103	€14	Gamma (€56;€1.83)	REACH+
True negative patients****	€57	€5	Gamma (€112;€0.51)	REACH+
False negative patients	€117	€11	Gamma (€104;€1.12)	REACH+
Productivity costs				
True positive patients	€1,368	€205	Gamma (€44;€31)	REACH+
False positive patients	€961	€144	Gamma (€44;€22)	REACH+
True negative patients	€710	€107	Gamma (€44;€16)	REACH+
False negative patients	€1,691	€254	Gamma (€44;€38)	REACH+
**Costs per 3 months – 12-60 months**				
MTX	€38	€3	Gamma (€44;€0.41)	[3]
Biologic DMARD	€3,500	€525	Gamma (€44;€79)	[3]
Other sDMARD	€27	€2	Gamma (€192;€0.14)	tREACH++
Direct medical costs				
DAS28≤2.6	€105	€3	Gamma (€964;€0.11)	tREACH++
DAS28>2.6–≤3.2	€133	€8	Gamma (€287;€0.47)	tREACH++
DAS28>3.2	€190	€6	Gamma (€959;€0.20)	tREACH++
Productivity costs				
DAS28≤2.6	€493	€74	Gamma (€44;€11)	tREACH++
DAS28>2.6–≤3.2	€908	€136	Gamma (€44;€20)	tREACH++

**Parameter**	**Value**	**SE**	**Distribution (alpha;beta)**	**Source**
DAS28>3.2	€1,247	€187	Gamma (€44;€28)	tREACH^{++}
**Costs non-RA patients**				
12-60 months (for 4 years)	€5,600	€840	Gamma (€44;€126)	Estimate$^{\#}$
**Cost new diagnostic tests**				
MRI of both hands and feet	€756			[4]
B-cell test	€150			Estimate$^{\#}$
IL-6 test	€50			Estimate$^{\#}$
Genetic assay test	€750			Estimate$^{\#}$
**Utilities**				
True positive patients – baseline	0.600	0.015	Beta (626;417)	REACH^{+}
12 months	0.700	0.010	Beta (2,354;1,569)	Estimate$^{\#}$
False positive patients –	0.650		Beta (548;295)	REACH^{+}
baseline	0.700	0.016	Beta (1,471;490)	Estimate$^{\#}$
12 months		0.010		
True negative patients –	0.650		Beta (548;295)<br>Beta (882;156)	REACH^{+}
baseline	0.750	0.016		Estimate$^{\#}$
12 months		0.011		
False negative patients –	0.600		Beta (626;417)	REACH^{+}
baseline	0.550	0.015	Beta (2,942;2,942)	Estimate$^{\#}$
12 months		0.007		
**Patients not using a biological – 12-60 months**				
EQ-5D always >0.5 over time				
DAS28≤2.6	0.858	0.003	Beta (11,632;1,923)	tREACH^{++}
DAS28>2.6–≤3.2	0.815	0.006	Beta (3,387;770)	tREACH^{++}
DAS28>3.2	0.763	0.005	Beta (5,220;1,625)	tREACH^{++}

Parameter	Value	SE	Distribution (alpha;beta)	Source
EQ-5D at least 1 time period <0.5				
DAS28≤2.6	0.746	0.015	Beta* (634;216)	tREACH++
DAS28>2.6–≤3.2	0.674	0.027	Beta* (201;97)	tREACH++
DAS28>3.2	0.462	0.025	Beta* (188;219)	tREACH++
**Biological patients – 12-60 months**				
EQ-5D always >0.5 over time				
DAS28≤2.6	0.825	0.008	Beta (1,990;421)	tREACH++
DAS28>2.6–≤3.2	0.770	0.009	Beta (1,548;461)	tREACH++
DAS28>3.2	0.751	0.006	Beta (3,692;1,226)	tREACH++
EQ-5D at least 1 time period <0.5				
DAS28≤2.6	0.694	0.034	Beta** (124;55)	tREACH++
DAS28>2.6–≤3.2	0.623	0.049	Beta** (61;37)	tREACH++
DAS28>3.2	0.558	0.026	Beta** (199;157)	tREACH++
**Non-RA patients**				
12-60 months	0.750	0.010	Beta (1471;490)	Estimate#
**Diagnostic performance of tests**				
B-cell test				
Sensitivity	0.60			[5]
Specificity	0.90			[5]
IL-6 serum level test				
Sensitivity	0.70			[6]
Specificity	0.53			[6]
MRI of both hands and feet				

Parameter	Value	SE	Distribution (alpha;beta)	Source
Sensitivity	0.90			[7-9]
Specificity	0.60			[7-9]
Genetic assay test				
Sensitivity	0.40			[10]
Specificity	0.85			[10]
**Discount rates**				
Costs	4.0%			[11]
Health effects	1.5%			[11]

Notes: SE: standard error; DHA: Dutch Healthcare Authority; +REACH data on file, details about data collection can be found in Alves et al.[12]; ++tREACH data on file, details about data collection can be found in De Jong et al.[13]; *Beta distribution adjusted to allow values from -0.2 based on observed tREACH data; ** Beta distribution adjusted to allow values from -0.1 based on observed tREACH data; #Estimate based on expert opinion, for the SE data from REACH was used; ***Costs include diagnostic test costs (frequency laboratory tests and radiographics based on expert opinion; costs derived from the DHA tariffs), medical consultation time based on the Dutch manual for costing in economical evaluations[11]; †continuation of MTX at 12 month is the % of MTX users at 6 months that still use MTX at 12 months; ****True negative patients could use other sDMARDS due to other classifiable disease

References

[1] Aletaha D, Neogi T, Silman AJ, et al. Rheumatoid arthritis classification criteria: an American College of Rheumatology/European League Against Rheumatism collaborative initiative. Ann Rheum Dis 2010;69(9):1580–8.

[2] Vermeer M, Kievit W, Kuper HH, et al. Treating to the target of remission in early rheumatoid arthritis is cost-effective: results of the DREAM registry. BMC Musculoskelet Disord 2013;14:350.

[3] National Health Care Institute. http://www.medicijnkosten.nl/default.asp. Accessed 2 February 2015.

[4] Dutch Healthcare Authority (in Dutch: Nederlandse Zorgauthoriteit, NZa). NZa Zorgproducten Tariefapplicatie. Available at: http://dbc-zorgproducten-tarieven.nza.nl/nzaZpTarief/ZoekfunctieDbc.aspx. Accessed May 1, 2013.

[5] Van Baarsen LG, Bos WH, Rustenburg F, et al. Gene expression profiling in autoantibody-positive patients with arthralgia predicts development of arthritis. Arthritis Rheum 2010;62(3):694-704.

[6] Gottenberg JE, Dayer JM, Lukas C, et al. Serum IL-6 and IL-21 are associated with markers of B cell activation and structural progression in early rheumatoid arthritis: results from the ESPOIR cohort. Ann Rheum Dis. 2012;71(7):1243-8.

[7] Duer-Jensen A, Hørslev-Petersen K, Hetland ML, et al. Bone edema on magnetic resonance imaging is an independent predictor of rheumatoid arthritis development in patients with early undifferentiated arthritis. Arthritis Rheum. 2011;63(8):2192-202.

[8] Bird P, Conaghan P, Ejbjerg B, et al. The development of the EULAR-OMERACT rheumatoid arthritis MRI reference image atlas. Ann Rheum Dis 2005;64(1):i8-10.

[9] Nieuwenhuis WP, Krabben A, Stomp W, et al. Evaluation of magnetic resonance imaging-detected tenosynovitis in the hand and wrist in early arthritis. Arthritis Rheumatol 2015;67(4):869-76.

[10] Yarwood A, Huizinga TW, Worthington J. The genetics of rheumatoid arthritis: risk and protection in different stages of the evolution of RA. Rheumatology 2014 (in press).

[11] College voor zorgverzekeringen (CVZ). Guidelines for pharmacoeconomic research, updated version. Diemen: College voor zorgverzekeringen 2006.

[12] Alves C, Luime JJ, Van Zeben D, et al. Diagnostic performance of the ACR/EULAR 2010 criteria for rheumatoid arthritis and two diagnostic algorithms in an early arthritis clinic (REACH). Ann Rheum Dis 2011;70(9):1645–7.

[13] De Jong PH, Hazes JM, Han HK, et al. Randomised comparison of initial triple DMARD therapy with methotrexate monotherapy in combination with low-dose glucocorticoid bridging therapy; 1-year data of the tREACH trial. Ann Rheum Dis 2014;73(7):1331–9.
